# Primary Tumor Resection Plus Chemotherapy versus Chemotherapy Alone for Colorectal Cancer Patients with Synchronous Bone Metastasis

**DOI:** 10.3390/medicina59081384

**Published:** 2023-07-28

**Authors:** Yanqing Li, Xiaofei Cheng, Chenhan Zhong, Ying Yuan

**Affiliations:** 1 Department of Pathology, The Second Affiliated Hospital, Zhejiang University School of Medicine, Hangzhou 310009, China; yanqingli@zju.edu.cn; 2 Department of Colorectal Surgery, The First Affiliated Hospital, Zhejiang University School of Medicine, Hangzhou 310003, China; xfcheng@zju.edu.cn; 3 Department of Medical Oncology, The Second Affiliated Hospital, Zhejiang University School of Medicine, Hangzhou 310009, China; chenhanzhong@zju.edu.cn

**Keywords:** colorectal cancer (CRC), bone metastasis (BM), survival, chemotherapy, primary tumor resection (PTR)

## Abstract

*Background and Objective*: Colorectal cancer (CRC) bone metastasis (BM), particularly synchronous metastasis, is infrequent and has a poor prognosis. Radical surgery for CRC with BM is challenging, and chemotherapy is the standard treatment. However, it is unclear whether combining chemotherapy with primary tumor resection (PTR) yields greater survival benefits than chemotherapy alone, as no relevant reports exist. *Material and Methods*: The Surveillance, Epidemiology, and End Results (SEER) database provided data on 1662 CRC patients with bone metastasis between 2010 and 2018, who were divided into two groups: chemotherapy combined with PTR and chemotherapy alone. Survival distributions were compared using the log-rank test, and survival estimates were obtained using the Kaplan–Meier method. A Cox proportional multivariate regression analysis was conducted to estimate the survival benefit of chemotherapy combined with PTR while controlling for additional prognostic factors. *Results*: The chemotherapy only group consisted of 1277 patients (76.8%), while the chemotherapy combined with PTR group contained 385 patients (23.2%). Patients who received chemotherapy combined with PTR had a significantly higher 1-year survival rate (60.7%) and 2-year survival rate (32.7%) compared to those who only received chemotherapy (43.8% and 18.4%, respectively; *p* < 0.0001). Independent prognostic factors identified by Cox proportional analysis were age, location of the primary tumor, type of tumor, M stage, metastasectomy and PTR. Patients who received chemotherapy combined with PTR had a significantly improved prognosis (HR 0.586, 95% CI 0.497–0.691, *p* < 0.0001). All subgroups demonstrated a survival advantage for patients who received chemotherapy in combination with PTR. *Conclusions*: Our findings suggest that patients with BM from CRC may benefit from chemotherapy combined with PTR. Our analysis also identified age, location of the primary tumor, type of tumor, M stage, metastasectomy, and PTR as independent prognostic risk factors for CRC patients with synchronous BM.

## 1. Introduction

Colorectal cancer (CRC) is the third most common cancer globally and the leading cause of cancer-related deaths in both males and females [1]. Distant metastasis is a major contributor to the poor prognosis of individuals with CRC. Although CRC typically metastasizes to the liver and lungs more frequently than to other organs, bone metastasis is relatively rare. Research suggested that the prevalence of bone metastasis in individuals with CRC may range from 1.2% to 12% [2,3]. However, the number of cases of bone metastasis may be increasing due to advances in early detection of CRC, closer monitoring, and better patient survival [4]. Patients with bone metastasis (BM) from CRC have a significantly poor survival outcome, with a median life expectancy of between 7 and 9.4 months [5,6]. Furthermore, various skeletal-related events (SREs), such as malignant hypercalcemia, pathological fractures, and spinal cord compression, have a significant negative impact on the quality of life of affected individuals [7]. Therefore, CRC BM should not be overlooked as a minor clinical issue that requires little consideration.

Chemotherapy is the standard treatment for metastatic colorectal cancer, but the role of primary tumor resection (PTR) in individuals with asymptomatic primary tumors and inoperable metastatic disease is debatable. While some studies suggest that PTR can improve long-term survival, others argue that the risks and complications associated with the procedure may outweigh any potential benefits, especially if chemotherapy is delayed [8,9,10]. Two randomized controlled trials have failed to demonstrate a significant improvement in survival with PTR [11,12], but a meta-analysis has shown that it can be a predictor of prolonged life for some individuals with metastatic colorectal cancer [13]. However, there is currently no consensus on the use of PTR in combination with chemotherapy for this patient population. The impact of PTR on individuals with CRC BM is also unclear, given the low incidence of this complication.

This study aimed to investigate whether the combination of PTR and chemotherapy improves survival in patients with synchronous CRC BM, as compared to those receiving chemotherapy alone. To our knowledge, this is the first study to examine the impact of PTR on the survival of CRC BM patients.

## 2. Material and Methods

### 2.1. Study Population

The study population consisted of individuals newly diagnosed with CRC between the years 2010 and 2018, as obtained from the Surveillance, Epidemiology, and End Results (SEER) database. A total of 339,204 CRC patients were included in the database during this period. Among them, 4138 patients were identified to have concurrent bone metastases at the time of CRC diagnosis, which were classified as synchronous bone metastasis.

**Figure 1:**
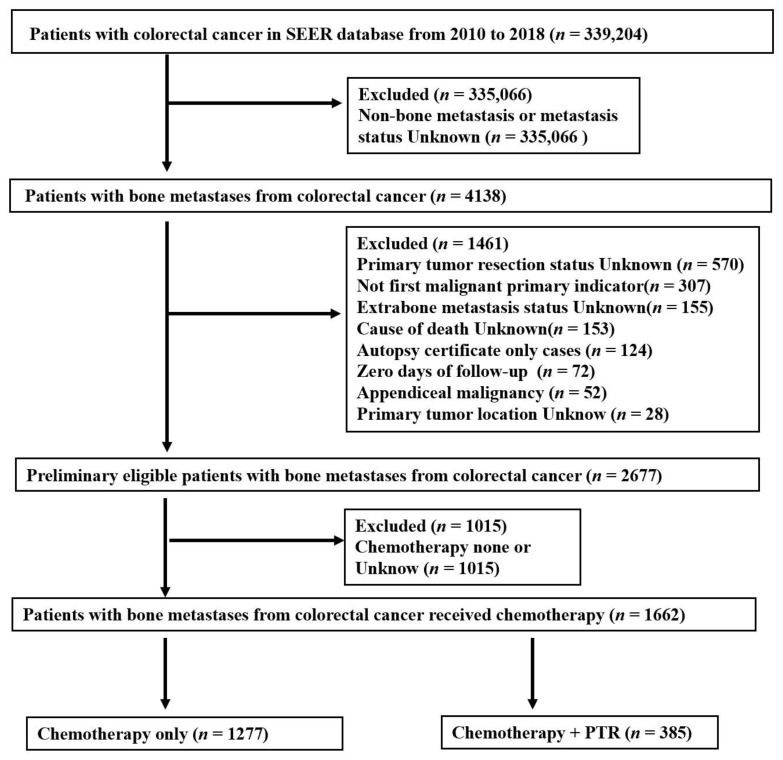
Flow diagram illustrating the selection process of eligible colorectal cancer (CRC) patients with bone metastasis (BM) from the SEER database, categorized into two groups: Chemotherapy alone and chemotherapy combined with primary tumor resection (Chemotherapy + PTR). The figure depicts the sequential steps of patient inclusion and exclusion based on specific criteria, leading to the final study cohorts for each group.

For the clinicopathological analysis, various factors were considered, including age (younger than 60 or 60 and above), gender, histology type (adenocarcinoma or other), primary site surgery, carcinoembryonic antigen (CEA) status, T stage, N stage, M stage, radiation treatment, metastasectomy, cancer-specific survival (CSS), and survival time [14]. To classify the primary tumor location in CRC, the anatomical subtypes were taken into account. Right-sided colon cancer (RCC) referred to tumors located from the cecum to the transverse colon, left-sided colon cancer (LCC) referred to tumors located from the splenic flexure to the sigmoid colon, and rectal cancer (RC) referred to tumors located from the rectosigmoid junction to the rectum [14]. The research team tracked the progress of the patients from the time of CRC diagnosis until the end of the trial, their death, or the last follow-up, whichever occurred first. The selection process of the participants is detailed in Figure 1.

After a thorough screening of the collected data, certain cases were excluded from the analysis. This included 28 cases with unknown primary tumor location, 153 cases with unknown cause of death, 124 cases diagnosed through autopsy, 307 cases with a history of other malignant tumors prior to CRC diagnosis, 72 cases with missing follow-up time, 52 cases of appendiceal tumors, 520 cases with unknown information on primary tumor surgery, and 155 cases with unknown information on metastasis to other sites apart from bone, resulting in a total exclusion of 1461 cases.

The remaining 2677 cases underwent further analysis. Among these, 1015 cases either did not receive chemotherapy or had missing information regarding chemotherapy. Therefore, the final number of patients included in the effective analysis was 1662. Among these, 1277 patients received chemotherapy alone, while 385 patients received a combination of chemotherapy and primary tumor resection.

It is important to note that due to the nature of the SEER database, specific information regarding targeted therapy was not available for inclusion in this study.

The detailed selection process and the remaining number of patients for analysis are summarized in Figure 1.

### 2.2. Statistical Analysis

Statistical analyses were performed using SPSS version 25 and GraphPad Prism 8. The association between categorical variables and numerical data, expressed as percentages, was assessed using the Chi-square test and Fisher’s exact test. Survival curves were generated using the Kaplan–Meier method and compared using the log-rank test. Cox proportional hazards models were employed to identify prognostic factors for patients with bone metastases, and a multivariate analysis was conducted to determine independent predictors of prognosis. Variables with a *p*-value less than 0.05 were considered statistically significant. The study cohort consisted of a total of 1662 patients with bone metastasis from colorectal cancer.

To ensure the reliability of our findings, rigorous data collection procedures were implemented. The SEER database, renowned for its comprehensive coverage and reliable information, provided a valuable resource for this study. By utilizing this database, we were able to gather a substantial sample size, encompassing a wide range of clinicopathological factors and long-term survival outcomes. This allowed for robust statistical analyses, enhancing the validity and generalizability of our results. A comprehensive analysis of various clinicopathological factors and survival outcomes was conducted. Statistical analyses were performed using established software, and stringent selection criteria were employed. The utilization of the SEER database and the implementation of rigorous data analysis methods contribute to the strength and reliability of this study.

## 3. Results

### 3.1. Patient Characteristics

Among the 339,204 patients with CRC diagnosed between 2010 and 2018, a total of 4138 individuals were identified to have synchronous BM, corresponding to a rate of 1.22%. After a comprehensive assessment, our study population consisted of 1662 CRC patients with bone metastases (Figure 1). Within this cohort, 385 patients (23.2%) received a combination of chemotherapy and PTR, while 1277 patients (76.8%) received chemotherapy alone. Notable differences were observed between the two groups in terms of age, primary tumor location, T-stage, N-stage, M-stage, radiotherapy, metastasectomy, and CEA levels. The chemotherapy-only group had a substantial proportion of patients with unclear T-stage, N-stage, or M-stage, which accounted for a significant portion of the overall group. In contrast, the chemotherapy/PTR combination group had a higher proportion of patients with RCC, radiation therapy, age over 60, and negative CEA status. Among the 1662 individuals with synchronous bone metastases, the majority (82.8%, *n* = 1376) also had metastases outside the bone. Detailed patient characteristics are presented in Table 1.

### 3.2. Survival and Prognostic Factors

**Figure 2:**
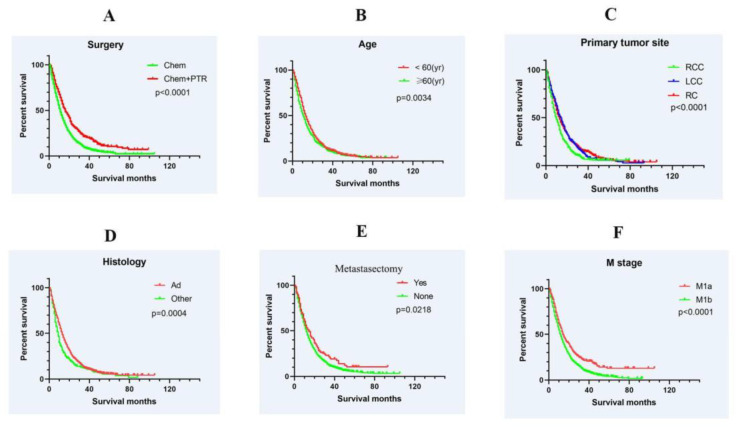
Kaplan–Meier survival curve analysis for cancer-specific survival (CSS) in colorectal cancer (CRC) patients with synchronous bone metastasis (BM). The analysis was performed based on various factors, illustrating the differences in survival outcomes between different patient subgroups. (**A**) Comparison between the Chemotherapy + PTR group and the Chemotherapy alone group, showing a superior prognosis in the Chemotherapy + PTR group. (**B**) Comparison of patients aged 60 years and above with those under 60 years, indicating a poorer prognosis in the older age group. (**C**) Comparison of patients with primary tumors located in the rectum and left colon with those in the right colon, demonstrating a better prognosis in the rectum and left colon groups. (**D**) Comparison of patients with adenocarcinoma with those with other histological types, revealing a better prognosis in the adenocarcinoma group. (**E**) Comparison of patients with BM treated with bone metastasis excision with those without bone metastasis excision, showing a survival advantage in the bone metastasis excision group. (**F**) Comparison of patients with concurrent bone and extra-bone metastases with those with bone metastases alone, indicating a poorer prognosis in the group with concurrent metastases. These findings highlight the impact of different factors on the survival outcomes of CRC patients with synchronous bone metastasis.

Out of the 2677 individuals diagnosed with colorectal cancer and synchronous bone metastases, 1662 received chemotherapy, while the remaining 1015 did not. The median survival time for patients who received chemotherapy was 12 months, whereas those who did not receive chemotherapy had a significantly shorter median survival time of only 2 months. Further analysis was conducted within the chemotherapy group, dividing patients into two subgroups: those who received chemotherapy alone and those who underwent chemotherapy combined with PTR. The group receiving chemotherapy combined with PTR demonstrated a 1-year survival rate of 60.7% and a 2-year survival rate of 32.7%, which were significantly higher than the rates of 43.8% and 18.4% observed in the chemotherapy-only group (*p* < 0.0001; Figure 2A). The median survival time for the chemotherapy/PTR group was 16 months, compared to 11 months for the chemotherapy-only group. Stratifying by age, patients under 60 years old had a median survival time of 13 months, while those over 60 had a median survival time of 10 months. The difference in cancer-specific survival (CSS) between the two age groups was statistically significant (*p* = 0.0023; Figure 2B).

Regarding the primary tumor location, the 1-year survival rate for LCC and RC was significantly higher than that for RCC, with rates of 52.7% and 50.0% compared to 40.5%, respectively (*p* < 0.0001; Figure 2C). Histologically, patients with adenocarcinoma had a median survival time of 13 months, while those with other histologies had a median survival time of 9 months (*p* = 0.0004; Figure 2D). The group that underwent metastasectomy had a 1-year survival rate of 53.4%, which was higher than the rate of 47.3% observed in the non-metastasectomy group (*p* = 0.0218; Figure 2E). Patients without metastases outside the bone experienced a greater survival benefit compared to those with metastases beyond the bone (*p* < 0.0001; Figure 2F).

Univariate analysis identified age, CEA level, primary tumor site, histology, M stage, radiotherapy, metastasectomy, and metastases outside the bone as factors affecting the CSS of CRC patients with synchronous bone metastases (Table 2). No statistically significant correlation was observed between sex, T stage, and N stage. Multivariate analysis, considering these important factors, revealed that age, primary tumor location, histology, M stage, metastasectomy, and PTR were independent predictors of outcome (Table 3). Older patients had a lower CSS rate compared to younger patients (HR 1.171, 95% CI 1.047–1.309, *p* = 0.006). The CSS rate was significantly higher among patients who underwent chemotherapy combined with PTR compared to those who did not undergo PTR (HR 0.586, 95% CI 0.497–0.691, *p* < 0.0001).

**Figure 3:**
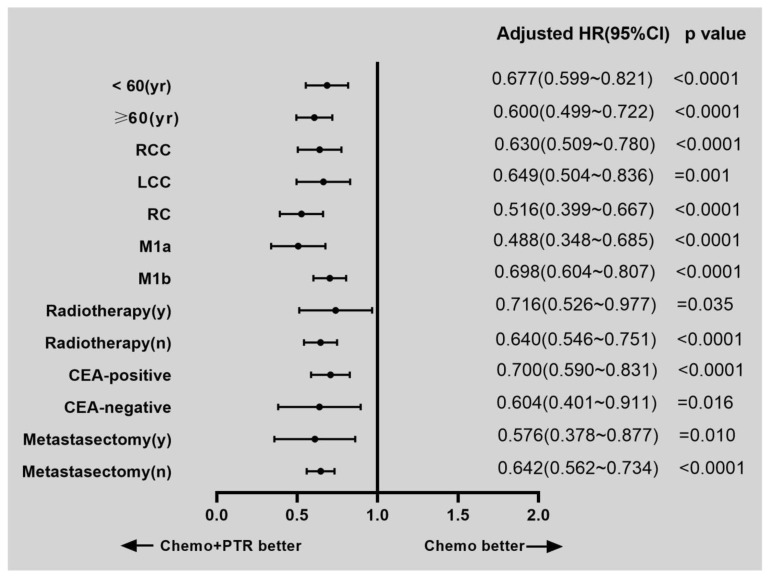
The adjusted hazard ratios (HRs) for cancer-specific survival (CSS) were calculated from a comprehensive subgroup analysis, encompassing multiple parameters, including age, location of the primary tumor, M stage, radiotherapy, CEA level, and metastasectomy. The analysis revealed that in all subgroups, the Chemotherapy + PTR group achieved a survival advantage compared to the Chemotherapy alone group.

To address any potential confounding factors between the chemotherapy/PTR and chemotherapy-only groups, additional subgroup analyses were conducted. The adjusted hazard ratios for CSS varied based on age, primary tumor location, M stage, radiotherapy, metastasectomy, and CEA level (Figure 3). In all subgroups, patients who received chemotherapy combined with PTR demonstrated a survival advantage.

In summary, our results demonstrate that chemotherapy combined with PTR was associated with improved survival outcomes in CRC patients with synchronous bone metastases. Age, primary tumor location, histology, M stage, metastasectomy, and PTR were identified as independent prognostic factors. These findings highlight the importance of a multimodal treatment approach for this patient population and support the consideration of PTR as a therapeutic option in combination with chemotherapy.

## 4. Discussion

Chemotherapy is commonly employed in advanced-stage colorectal cancer to alleviate symptoms, manage cancer progression, and extend patient survival [15]. Our study revealed that patients who underwent chemotherapy had a median survival of 12 months, whereas those who did not receive treatment had only 2 months of survival. Despite the benefits of chemotherapy, the management of metastatic colorectal cancer remains a complex challenge. In recent years, targeted therapy has emerged as a crucial approach in treating various cancers, including colorectal cancer bone metastasis [16]. Targeted therapy is specifically aimed at the molecular alterations or specific pathways involved in cancer growth and spread, leading to more effective and tailored treatments [17,18]. Although the SEER database lacks information on targeted therapy, it is essential to acknowledge its significant role in the treatment of colorectal cancer bone metastasis. The addition of primary tumor resection (PTR) to chemotherapy for asymptomatic patients has been a topic of intense debate compared to chemotherapy alone [9]. While some retrospective investigations have suggested that resecting the primary tumor may lead to increased survival rates [13,19], a recent randomized controlled trial (RCT) has disputed this claim [11]. This prompted our study to focus on the specific site of metastasis, namely, colorectal cancer bone metastasis, as previous research has not distinguished between various metastatic sites. 

Our study, utilizing the SEER database, sought to address the limitation of the low incidence rate of concurrent colorectal cancer bone metastasis. The findings demonstrated that in patients with colorectal cancer bone metastasis, PTR combined with chemotherapy significantly improved survival compared to chemotherapy alone. Subgroup analyses based on age, primary tumor site, CEA level, metastasectomy, and M-stage further supported the survival benefit of PTR combined with chemotherapy over chemotherapy alone.

To shed light on the potential mechanisms underlying the survival benefit, we speculate that PTR may reduce tumor burden, thereby lessening the tumor’s impact on the body and extending survival [20]. Additionally, the combination of PTR with chemotherapy might exert further control over the growth of other metastatic lesions, contributing to improved patient outcomes [21]. Further investigation into the mechanisms involved, such as the potential modulation of the immune system or changes in the tumor microenvironment, is essential to provide a more comprehensive understanding of the benefits of PTR combined with chemotherapy for colorectal cancer patients with bone metastasis. Overall, our study suggests that considering PTR as part of the treatment strategy for colorectal cancer patients with bone metastasis could have a positive impact on their prognosis and overall survival. Nonetheless, more research is needed to elucidate the precise mechanisms behind these findings, potentially opening doors to more effective and personalized treatment approaches.

The findings of our study highlight age as a significant determinant of bone metastases prognosis in CRC. Patients were categorized into two age groups: under 60 and over 60 years old, with those under 60 demonstrating a more favorable prognosis. Similar conclusions have been observed in patients with colorectal cancer metastasis in other sites, suggesting that the better prognosis in younger patients may be attributed to their superior physical condition and higher tolerance for aggressive treatments [19]. Mechanistic investigations can shed light on the underlying factors contributing to age-related differences in prognosis. Younger patients may exhibit a more active immune system, enabling better suppression of tumor progression [22,23]. Additionally, age-related variances in tumor biology may influence treatment responses and affect prognosis [23,24]. It is conceivable that younger patients could display enhanced treatment sensitivity, leading to improved therapeutic outcomes. Considering the impact of age on prognosis, clinical implications emerge. For younger colorectal cancer patients with bone metastasis, a more proactive treatment approach could be considered, including the adoption of potent chemotherapy regimens and surgical resection when feasible. In contrast, older patients may require a more cautious selection of treatment modalities, especially if they have coexisting chronic conditions or compromised physical health, necessitating a balanced assessment of risks and benefits.

Our study also found that in patients with simultaneous CRC BM, those with primary tumors located in the left colon had better prognosis than those with tumors located in the right colon. Similar to previous studies, metastatic colorectal cancer with primary tumors located in the right colon tends to have a poorer prognosis than those located in the left colon [25,26,27,28]. This may be due to biological differences between the two regions, such as differences in blood supply and genetic stability [25,29]. However, further research is needed to fully understand the underlying mechanisms behind this observed difference. Nonetheless, our results highlight the importance of considering primary tumor location when determining the best treatment strategy and prognostic assessment for patients with colorectal cancer and bone metastasis.

In addition, our study found that extraosseous metastasis, treatment of bone metastatic lesions, and histological type (adenocarcinoma versus other types) were also independent risk factors affecting the prognosis of synchronous colorectal cancer bone metastasis. A previous study by Svensson’s team [30] showed that rectal cancer patients with only bone metastasis had a median survival of 114 days, while those with bone and other organ metastases had a median survival of 79 days (95% CI 1.06–2.05). Colon cancer patients had a median survival of 105 days for bone metastasis and 95 days for bone and organ metastases (95% CI 1.02–1.87). Multiple metastases often indicate advanced disease and poor prognosis in colorectal cancer. Studies suggest that compared to single-site metastases, multiple metastases may be more difficult to treat and result in shorter survival time for patients [31]. This may be due to the more aggressive and complex nature of the tumor with multiple metastases, making it challenging to control its growth and spread through local treatments such as surgery or radiation therapy [17]. Adenocarcinoma, the most common type of colorectal cancer, typically has a better prognosis than other pathological types due to its better degree of differentiation and tissue structure, and higher likelihood to respond to chemotherapy and radiotherapy [32,33]. Therefore, among patients with synchronous colorectal cancer bone metastasis, those with adenocarcinoma pathology often have a better prognosis. These findings highlight the importance of considering multiple factors when evaluating the prognosis and treatment options for patients with CRC BM.

Our study has several limitations that need to be acknowledged. Firstly, we only included patients with synchronous colorectal cancer and bone metastasis due to the SEER database’s limitation in recording bone metastasis status at the time of initial diagnosis. This exclusion may affect the generalizability of our findings to patients with metachronous bone metastasis. Secondly, the lack of clinicopathological factors and genetic information, such as RAS/BRAF/MSI status, in the SEER database could introduce bias and limit the accuracy of our analysis. The absence of these critical genetic markers may have implications for treatment decisions and could impact the overall outcomes for patients with colorectal cancer and bone metastasis [34]. Additionally, our study did not explore specific details regarding the types and regimens of chemotherapy drugs utilized by the patients. Understanding the specific chemotherapy protocols could provide valuable insights into treatment responses and efficacy for different patient subgroups. Furthermore, we did not investigate the use of targeted therapies in our study. Targeted therapies play a significant role in the treatment of advanced colorectal cancer and may have affected patient outcomes [16]. Examining the utilization and efficacy of targeted therapies could have provided valuable information for a more comprehensive analysis [35]. Lastly, we did not address the topic of palliative care for bone metastases in colorectal cancer patients. Considering the importance of palliative treatments, such as radiotherapy and pain management, in improving the quality of life for patients with bone metastasis, future research should explore these aspects. Despite these limitations, our study offers valuable insights into the prognostic significance of primary tumor resection combined with chemotherapy in colorectal cancer patients with bone metastasis. Further investigations incorporating more comprehensive data sources and genetic information are warranted to validate and expand upon our findings.

In conclusion, compared to chemotherapy alone, combined chemotherapy and PTR can provide better survival benefits for patients with synchronous CRC BM. Age, primary tumor location, histological type, extraosseous metastasis, bone metastasis lesion treatment, and PTR are independent risk factors for the prognosis of synchronous CRC BM. It is important to consider these factors in the management and treatment of patients with CRC BM. With further research and understanding of the underlying mechanisms, more effective treatment strategies can be developed to improve the prognosis and quality of life for these patients.

## Figures and Tables

**Table 1:** Baseline characteristics of patients in the chemotherapy group and the chemotherapy combined with PTR group.

Variable	Total (*n* = 1662)		Chemo (*n* = 1277)		Chemo + PTR (*n* = 385)		*p* Value
Age (yr)							0.007
<60	852	(51.3)	678	(53.1)	174	(45.2)	
≥60	810	(48.7)	599	(46.9)	211	(54.8)	
Gender							0.409
Female	665	(40.0)	504	(39.5)	161	(41.8)	
Male	997	(60.0)	773	(60.5)	224	(58.2)	
Primary tumor site							<0.0001
RCC	499	(30.0)	330	(25.8)	169	(43.9)	
LCC	404	(24.3)	285	(22.3)	119	(30.9)	
RC	759	(45.7)	662	(51.8)	97	(25.2)	
Histology							0.856
Adenocarcinoma	1395	(83.9)	1073	(84.0)	322	(83.6)	
Other	267	(16.1)	204	(16.0)	63	(16.4)	
T stage							<0.0001
T0/T1	178	(10.7)	171	(13.4)	7	(1.8)	
T2	35	(2.1)	25	(2.0)	10	(2.6)	
T3	379	(22.8)	219	(17.1)	160	(41.6)	
T4	387	(23.3)	207	(16.2)	180	(46.8)	
Tx	683	(41.1)	655	(51.3)	28	(7.3)	
N stage							<0.0001
N0	505	(30.4)	454	(35.6)	51	(13.2)	
N1	489	(29.4)	372	(29.1)	117	(30.4)	
N2	315	(19.0)	109	(8.5)	206	(53.5)	
Nx	353	(21.2)	342	(26.8)	11	(2.9)	
M stage							
M1a	286	(17.2)	206	(16.1)	80	(20.8)	<0.0001
M1b	1376	(82.8)	1071	(83.9)	305	(79.2)	
Radiotherapy							
Yes	216	(13.0)	83	(6.5)	133	(34.5)	<0.0001
None	1446	(87.0)	1194	(93.5)	252	(65.5)	
CEA							
Positive	931	(56.0)	730	(57.2)	201	(52.2)	<0.0001
Negative	133	(8.0)	83	(6.5)	50	(13.0)	
Unknown	598	(36.0)	464	(36.3)	134	(34.8)	
Metastasectomy							
Yes	127	(7.6)	76	(6.0)	51	(13.2)	<0.0001
None	1535	(92.4)	1201	(94.0)	334	(86.8)	

Abbreviations: PTR, primary tumor resection; RCC, right colon cancer; LCC, left colon cancer; RC, rectal cancer.

**Table 2:** Univariate Cox regression for cancer specific survival among patients with bone metastases received chemotherapy.

Clinicopathologic Variable	HR	95% CI	*p* Value
Age (yr)			
<60	Reference		
≥60	1.186	1.063–1.323	0.002
Gender			
Female	Reference		
Male	0.963	0.861–1.076	0.503
Primary tumor site			
RCC	Reference		
LCC	0.741	0.638–0.860	<0.0001
RC	0.742	0.653–0.843	<0.0001
Histology			
Adenocarcinoma	Reference		
Other	1.294	1.118–1.496	0.001
T stage			
T0/T1	Reference		
T2	0.774	0.522–1.147	0.202
T3	0.700	0.572–0.855	<0.0001
T4	0.846	0.694–1.032	0.099
Tx	0.943	0.786–1.131	0.527
N stage			
N0	Reference		
N1	0.989	0.860–1.137	0.875
N2	1.111	0.950–1.300	0.188
Nx	1.198	1.019–1.408	0.029
M stage			
M1a	Reference		<0.0001
M1b	1.444	1.240–1.682	
Radiotherapy			
Yes	Reference		0.003
None	1.278	1.085–1.504	
CEA			
Positive	Reference		
Negative	0.556	0.406–0.759	<0.0001
Unknown	1.167	0.965–1.410	0.110
Surgery			
Non-PTR	Reference		
PTR	0.642	0.562–0.734	<0.0001
Metastasectomy			
Yes	Reference		
None	1.297	1.052–1.598	0.015

Abbreviations: PTR, primary tumor resection; RCC, right colon cancer; LCC, left colon cancer; RC, rectal cancer; CI, confidence interval; HR, hazard ratio.

**Table 3:** Multivariable Cox regression for cancer specific survival (CSS) among patients with bone metastases received chemotherapy.

Clinicopathologic Variable	HR	95% CI	*p* Value
Age (yr)			
<60	Reference		
≥60	1.171	1.047–1.309	0.006
Primary tumor site			
RCC	Reference		
LCC	0.750	0.644–0.874	<0.0001
RC	0.694	0.605–0.795	<0.0001
Histology			
Adenocarcinoma	Reference		
Other	1.333	1.148–1.548	<0.0001
M stage			
M1a	Reference		
M1b	1.425	1.220–1.664	<0.0001
Metastasectomy			
Yes	Reference		
None	1.284	1.015–1.623	0.037
Surgery			
Non-PTR	Reference		
PTR	0.586	0.497–0.691	<0.0001

Abbreviations: PTR, primary tumor resection; RCC, right colon cancer; LCC, left colon cancer; RC, rectal cancer; CI, confidence interval; HR, hazard ratio.

## Data Availability

The data that support the findings of this study are openly available in the Surveillance, Epidemiology, and End Results (SEER) database of the National Cancer Institute at https://seer.cancer.gov/ (accessed on 22 August 2022).
